# Age-dependent alteration of antioxidant defense system in hypertensive and type-2 diabetes patients

**DOI:** 10.1186/s40200-015-0164-z

**Published:** 2015-04-21

**Authors:** Stanley IR Okoduwa, Ismaila A Umar, Sani Ibrahim, Fatima Bello, Nathan Habila

**Affiliations:** Infohealth Awareness Unit, SIRONigeria Global Limited, Abuja, Nigeria; Department of Biochemistry, Ahmadu Bello University, Zaria, Nigeria; Department of Medicine, Ahmadu Bello University Teaching Hospital, Shika, Nigeria; Department of Biochemistry and Molecular Biology, Monash University Clayton, Melbourne, VIC 38000 Australia

**Keywords:** Antioxidants, Aging, Oxidative stress, Diabetes and Hypertension

## Abstract

**Background:**

The association between hypertension and diabetes has been linked to increased oxidative stress with age. This study was to examine the level of age-dependent alterations in antioxidant defense system between patients having hypertension and/or type-2 diabetes.

**Methods:**

The study was conducted at the Ahmadu Bello University Teaching Hospital, Zaria-Nigeria, using 200 Subjects recruited from the cardiology, endocrinology and outpatient clinics. They were divided into four groups of 50 subjects each, namely: Diabetic group (DG), hypertensive (HG) and hypertensive-diabetic group (HDG) as cases. The control group (CG) was non-diabetic normotensive subjects. They were all stratified into six age-ranges namely 20–29, 30–39, 40–49, 50–59, 60–69, 70–79 years. Oxidative stress markers (lipid peroxidation, antioxidant vitamins and elements, enzymatic and non-enzymatic antioxidant) were measured in the blood sample collected from all subjects in each age group within the study groups.

**Results:**

The results in the DG, HG and HDG, showed that the percentage decrease in enzymatic antioxidants and antioxidant vitamins with age were significantly (P < 0.05) higher than 10.8% and 20.0% respectively when compared to the CG, whereas, the level of decrease in serum Selenium at same age range was significantly higher than 52.8%. The level of lipid peroxidation in the cases was observed to be significantly (P < 0.05) higher than 89.9% when compared to the mean reference values (2.94 ± 0.05 nmol/ml) of the CG at same age range. Also, the decrease levels of endogenous antioxidants were observed to be directly related to aging.

**Conclusion:**

The result obtained demonstrates the percentage age-dependent alteration in oxidative stress markers. The percentage decrease in the antioxidant levels during aging could be an explanation to the possible link, underlying the complication of type-2 diabetes and hypertension in this locale. Hence, antioxidants supplements may be useful in the management of the diseases during aging.

## Background

Hypertension and diabetes are one of the most serious disease threats all over the world that decreases the quality of human life. The indication and prevalence of other associated complications are increasing the rate of morbidity and mortality in most part of the third world developing countries [1-3]. Oxidative stress is known to be a component of molecular and cellular tissue damage mechanisms in a wide spectrum of human diseases including hypertension, diabetes [4-6] and increasing age [7-11]. These risk factors is a common feature that has plagued Nigeria which has a present population of 170 million people but according to the United Nations, the population of Nigeria will reach 440 million by 2050, this will make Nigeria the 3rd most populous country in the world [12]. Unfortunately, in the Northern part of Nigeria a large number of patients affected by this disease and its associated complications have unsatisfactory awareness and knowledge. Yet, more and more people die for ignorance of these complications.

An antioxidant is a molecule capable of slowing or preventing the oxidation of other molecules. In a biological system, oxidation reaction produces free radicals which in turn can initiate chain reactions that damage cells. Antioxidants provide the extra electrons to the free radicals and prevent them from causing damage to the body’s cells and tissues [10]. Cells are protected by antioxidant defense system: a highly complex biochemical organization that consists of numerous enzymes and a large number of scavenger molecules [6,13]. Antioxidant can block the oxidation process by neutralizing free radicals and in doing so, they become oxidized hence they have to be neutralized by other specific enzymes or antioxidant molecules [5,14].

Under normal physiological conditions, there is a critical balance in the generation of free radicals and antioxidant defense systems used by organisms to deactivate and protect themselves against free radical toxicity [13]. However, the rate of this damage increases during aging process, as the efficiency of antioxidative and repair mechanism decrease [9]. Alteration in the oxidant/antioxidant equilibrium creates a condition known as oxidative stress [13]. Based on the importance of changes in the oxidant to antioxidant ratio in relation to the development of pathological conditions, a measure of antioxidant capacity of the blood is used as an index of the antioxidant status of the system [6,15-17]. Several reports have implicates this disease condition to vary from age to age yet this is still a cause for debate [18,19]. Of particular interest to us are patients who have diabetes and/or hypertension that are attending the cardiology, endocrinology and general outpatient clinics at Ahmadu Bello University Teaching Hospital (ABUTH) Zaria, Nigeria. To our knowledge, this is the largest university in Nigeria and the second largest in Africa; it is therefore expedient to understand the age related level of oxidative stress status of the populace of this locale. In our previous study, we reported that, the relationship that exist between biomarkers of oxidative stress and trace mineral elements are the consequence of the changes in antioxidant defense system which resulted in the pathophysiologic mechanisms underlying the complication of type 2 diabetes and associated hypertension [7].

In this study, the level of serum lipid peroxidation (Malondialdehyde: MDA), serum antioxidants (reduced glutathione: GSH, superoxide dismutase: SOD, catalase: CAT), antioxidant vitamins (Vitamins C and E) and some serum antioxidant elements (zinc: Zn, copper: Cu, selenium: Se, and iron: Fe) in patients with diabetes, hypertension and both hypertension/diabetes at various age range was investigated along with that of healthy adults at same age range in order to ascertain the effect of age changes on the antioxidant.

## Methodology

### Subjects

The present study was conducted on 200 subjects selected consecutively on meeting a set of selection criteria. They were divided into 50 patients per group based on their disease condition. This comprises of persons with type 2 diabetes group (DG), hypertensive group (HG) and hypertension/diabetes group (HDG). They were recruited from three different clinics at the ABUTH, Zaria-Nigeria. These clinics are: The Cardiology, Endocrinology and General Outpatients Clinics. All the subjects were stratified into six classes based on their age range: 20–29, 30–39, 40–49, 50–59, 60–69, and 70–79 years. The groups with hypertension were selected based on the diagnostic criteria of the World Health Organisation-International society of Hypertension Guidelines for the management of hypertension: Systolic Blood Pressure (SBP) above 140 mmHg and Diastolic Blood Pressure (DBP) above 90 mmHg [20]. The group with diabetes was selected based on the diagnostic criteria established by the World Health Organisation of fasting plasma glucose (FPG) above 7.0 mmol/l, and/or 2-hours post-prandial plasma glucose above 11.1 mM [21]. Fifty Apparently healthy individuals who were not having diabetes nor hypertension were matched for age, sex and body mass index comprising staff and medical students of ABUTH, Zaria were recruited as the control group (CG).

### Ethical consideration

The purpose of the study was explained to all the subjects and a written informed consent was obtained from them. The study was ethically approved by the ethical committee of the hospital and in accordance with the Helsinki Declaration [22].

#### Selection criteria

##### Exclusion

Failure to give a written informed consent, present or past smokers, Patients who were having hepatic disease or taking lipid lowering drugs or antioxidant vitamin supplements, probucol, allopurinol, quinidine, disopyramide, or other drugs known for affecting serum lipid peroxidation and antioxidant values.

##### Inclusion

Patients diagnosed with hypertension and/or type-2 diabetes on their first attendance to the clinics at ABUTH. The control subjects were medically certified healthy.

#### Sample collection

After an overnight fasting of about 12 hours, an aliquot blood samples were collected from all subjects. Fractions of the blood sample collected were placed into coagulating tubes and allowed to clot to obtain the serum. The serum was stored at −20°C until required.

### Analyses of sample

GSH was determined spectrophotometrically at 412 nm according to the method described by Tietze [23]. The activity of SOD was measured at 560 nm according to the method describe by Martins *et al.*, [24]. CAT activity was measured spectrophotometrically at 240 nm according to the method described by Aebi, [25]. Lipid peroxidation in serum was estimated colorimetrically by measuring MDA using the modified method of Das *et al.* [8]. The concentration of Vitamin C was measured according to the method described by Omaye *et al.* [26] colorimetrically at 530 nm. The concentration of Vitamin E was estimated by the method of Desai [27] colorimetrically at 520 nm. Fasting Plasma Glucose (FPG) was determined according to the modified glucose-oxidase method described by Trinder, [28]. Concentration of Selenium in serum was determined by a flameless graphite furnace Atomic Absorption Spectrophotometer (AAS), (AA670G, Shimadzu, Japan). Serum Fe, Zn and Cu were determined using LABKIT reagent assay kit (Chemelex, S.A, Spain) according to the manufacturer’s instructions [29,30]. Serum Fe was measured spectrophotometrically at 562 nm as described by Perrotta [29]. Serum Zn and Cu were determined as described by Burtis [30].

### Statistical analysis

All statistical analyses were done using a computer software program, Statistical Package for the Social Sciences (SPSS Cary, NC, USA) version 20. Analysis of variance (ANOVA) was used to compare data from all groups. Pearson’s rank correlation was used to assess the relationships between the various parameters. Differences between mean were assessed by Duncan Multiple Range Test (DMRT). Data were expressed as mean ± standard error of mean. P-values < 0.05 were considered statistically significant.

## Results

The percentage of male to female ratio was 48:52. They were evenly distributed into different groups on the bases of age and sex as shown in Table 1. The mean age for each age range is also presented in the Table 1. It was observed that there were no cases recorded for hypertensive group within the age range 1 (20 – 29 years). There were no significant (P > 0.05) difference between in the mean age of all the cases with respect to the specific age range (Table 1).

**Table 1:** **Distribution of subjects into different groups on the bases of age and sex**

				**CG**	**DG**	**HG**	**HDG**
**Groups**	**Age Range (years)**	**Number of subjects**	**Percentage ratio (Male/Female)**	**Mean age (years)**			
1	20 – 29	18	50/50	28.4	27.3	-	26.0
2	30 – 39	35	45/55	37.6	32.1	33.7	32.4
3	40 - 49	45	44/56	47.4	42.6	45.3	43.0
4	50 - 59	50	46/54	56.9	53.4	54.7	52.3
5	60 - 69	40	55/45	67.3	63.8	63.2	62.5
6	70 - 79	12	50/50	77.0	72.0	71.1	70.3
***TOTAL***	***20 - 79***	***200***	***48/52***	***52.1***	***48.1***	***46.7***	***47.5***

**CG**: Control Group**; DG**: Group with type 2 diabetes; **HG**: Group with hypertension. **HDG**: Group with hypertension and type 2 diabetes.

In Table 2, we presented the percentage gender distribution, FPG, blood pressure, BMI, status of biomarkers of oxidative stress and serum concentrations of some trace mineral elements in cases and control group. The FPG was significantly (P < 0.05) higher in all the cases when compared to the CG. There were no significant (P > 0.05) difference in body BMI, SBP, DBP of the CG and DG groups. However, the BMI, SBP and DBP were significantly (P < 0.05) higher in HG and HDG when compared to the CG and DG. Although the lipid peroxidation level was not significantly (P > 0.05) different between the DG and HG groups, but they were significantly (P < 0.05) higher than the CG and lower than the HDG. The antioxidant enzymes (CAT, SOD), antioxidant vitamins (Vitamins C and E) and some trace mineral elements were significantly (P < 0.05) decreased in all the cases when compared to the control group. The activities of SOD and CAT were not significantly (P > 0.05) different between the DG and HG. Serum Cu was significantly (P < 0.05) higher in all the cases (DG, HG and HDG) when compared to the control group (CG). Serum Fe was higher in the HG than DG, HDG and CG, but there was no significant (P > 0.05) difference between serum Fe in HG, CG and DG groups (Table 2).

**Table 2:** **Percentage gender distribution, fasting plasma glucose, blood-pressure, body-mass-index, status of biomarkers of oxidative stress and serum concentrations of some trace elements in cases and control group**

	**CG**	**DG**	**HG**	**HDG**
**Percentage ratio (male/female)**	**50/50**	**44/56**	**48/52**	**50/50**
FPG (mmol/l)	4.80 ± 0.11^a^	9.29 ± 0.15^c^	5.53 ± 0.11^b^	9.93 ± 0.13^d^
SBP (mmHg)	115.80 ± 0.95^a^	119.4 ± 1.38^a^	165.12 ± 2.84^b^	168.16 ± 3.19^b^
DBP (mmHg)	73.10 ± 0.94^a^	74.20 ± 1.14^a^	101.82 ± 1.30^b^	103.20 ± 1.33^b^
BMI (kg/m^2^)	25.00 ± 0.26^a^	25.98 ± 0.75^a^	28.31 ± 0.75^b^	28.15 ± 0.77^b^
GSH (mmol/l)	0.68 ± 0.03^c^	0.56 ± 0.04^b^	0.55 ± 0.04^b^	0.44 ± 0.03^a^
MDA (nmol/ml)	2.94 ± 0.05 ^a^	5.58 ± 0.17^b^	5.64 ± 0.18^b^	6.46 ± 0.11^c^
SOD (U/gHb)	1729.9 ± 51.7^c^	1452.1 ± 32.8^a,b^	1509.9 ± 33.9^b^	1368.3 ± 36.1^a^
CAT (U/gHb)	172.56 ± 1.69^c^	149.20 ± 3.75^b^	153.93 ± 3.16^b^	139.97 ± 3.77^a^
Vit-C (μmol/l)	66.04 ± 0.90 ^c^	34.23 ± 1.53^b^	30.84 ± 0.84^a^	31.31 ± 0.97^a,b^
Vit-E (μmol/l)	17.58 ± 0.32^d^	14.06 ± 0.27^b^	13.11 ± 0.34^c^	12.20 ± 0.30^a^
Zn (μM)	15.55 ± 0.26^b^	14.79 ± 0.23^a,^	14.59 ± 0.29^a^	14.99 ± 0.22^a,b^
Cu (μM)	13.82 ± 0.14^a^	14.61 ± 0.17^b^	16.15 ± 0.14^d^	15.58 ± 0.16^c^
Fe (μM)	16.18 ± 0.42^b^	15.44 ± 0.35^a,b^	16.39 ± 0.39^b^	15.01 ± 0.39^a^
Se (μM)	1.63 ± 0.05^b^	0.77 ± 0.03^a^	0.68 ± 0.03^a^	0.70 ± 0.02^a^

The superscript (“a, b, c, d”) indicates significantly difference at p < 0.05 for the different study groups (CG, DG, HG and HDG). Values with same superscripts along the row indicate no significant difference (for the groups) at p < 0.05. Values are mean ± SEM of 50 replicate determinations.

**CG**: Control Group**; DG**: Group with type 2 diabetes; **HG**: Group with hypertension. **HDG**: Group with hypertension and type 2 diabetes; **FPG**: Fasting Plasma glucose; **SBP**: Systolic Blood Pressure; **DBP**: Diastolic Blood Pressure; **BMI**: Body Mass Index; **GSH**: Reduced glutathione; **MDA**: Malondialdehyde; **CAT**: Catalase; **Vit-C**: Vitamin C (Ascorbic Acid); **Vit-E**: Vitamin E (α-Tocopherol); **Zn:** Serum Zinc; **Cu**: Serum Copper; **Fe:** Serum Iron; **Se:** Serum Selenium.

The percentage level of alteration of biomarkers of oxidative stress is presented in Table 3. It was observed that the percentage decrease of GSH in the HG, DG and HDG when compared to the CG group were – 19.1%, − 17.6% and – 35.3% respectively. The percentage increase in lipid peroxidation (MDA) in the HG, DG and HDG groups as compared to the CG were + 91.8%, 89.9% and 119.7% respectively. The details in the level of alteration in the antioxidant enzymes, vitamins and serum trace mineral element are shown in the Table 3.

**Table 3:** **Percentage level of alteration in oxidative-stress markers and serum trace elements from the Control Group**

	**CG**	**HG (%)**	**DG (%)**	**HDG (%)**
GSH (mmol/l)	0.68 ± 0.03	- 19.1	- 17.6	- 35.3
MDA (nmol/ml)	2.94 ± 0.05	+ 91.8	+89.8	+119.7
SOD (U/gHb)	1729.9 ± 51.7	- 12.7	- 16.0	- 20.9
CAT (U/gHb)	172.56 ± 1.69	- 10.8	- 13.5	- 18.9
Vit-C (μmol/l)	66.04 ± 0.90	- 53.3	- 48.2	- 52.6
Vit-E (μmol/l)	17.58 ± 0.32	- 25.4	- 20.0	- 30.6
Zn (μM)	15.55 ± 0.26	- 6.2	- 4.9	- 3.6
Cu (μM)	13.82 ± 0.14	+16.9	+5.7	+12.7
Fe (μM)	16.18 ± 0.42	- 1.3	+4.6	- 7.2
Se (μM)	1.63 ± 0.05	- 58.3	- 52.8	- 57.1

CG: values are mean ± SEM of 50 replicate determinations, (+): Percentage increase from normal reference range; (−) : Percentage decrease from normal reference range.

**CG**: Control Group**; DG**: Group with type 2 diabetes; **HG**: Group with hypertension. **HDG**: Group with hypertension and type 2 diabetes ; **GSH**: Reduced glutathione; **MDA**: Malondialdehyde; **CAT**: Catalase; **Vit-C**: Vitamin C (Ascorbic Acid); **Vit-E**: Vitamin E (α-Tocopherol). **Zn:** Serum Zinc; **Cu**: Serum Copper; **Fe:** Serum Iron; **Se:** Serum Selenium.

**Figure 1:**
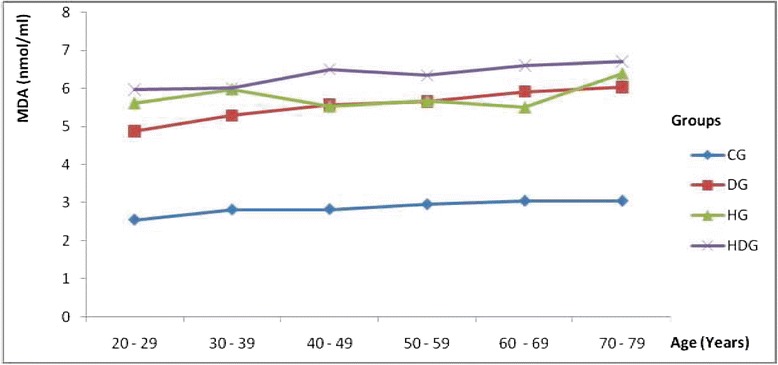
Changes in Lipid Peroxidation levels with age. **CG**: Control Group**; DG**: Group with type 2 diabetes; **HG**: Group with hypertension. **HDG**: Group with hypertension and type 2 diabetes.

It was observed that the pattern of lipid peroxidation production at different age range follow a different trend in all the cases when compared to the control group (Figure 1). The production of lipid peroxidation in the DG and HG group were observed to vary at different ages. But among the HDG group, the lipid peroxidation level was observed to be significantly (P < 0.05) higher than the observation recorded in the CG, DG and HG group.

**Figure 2:**
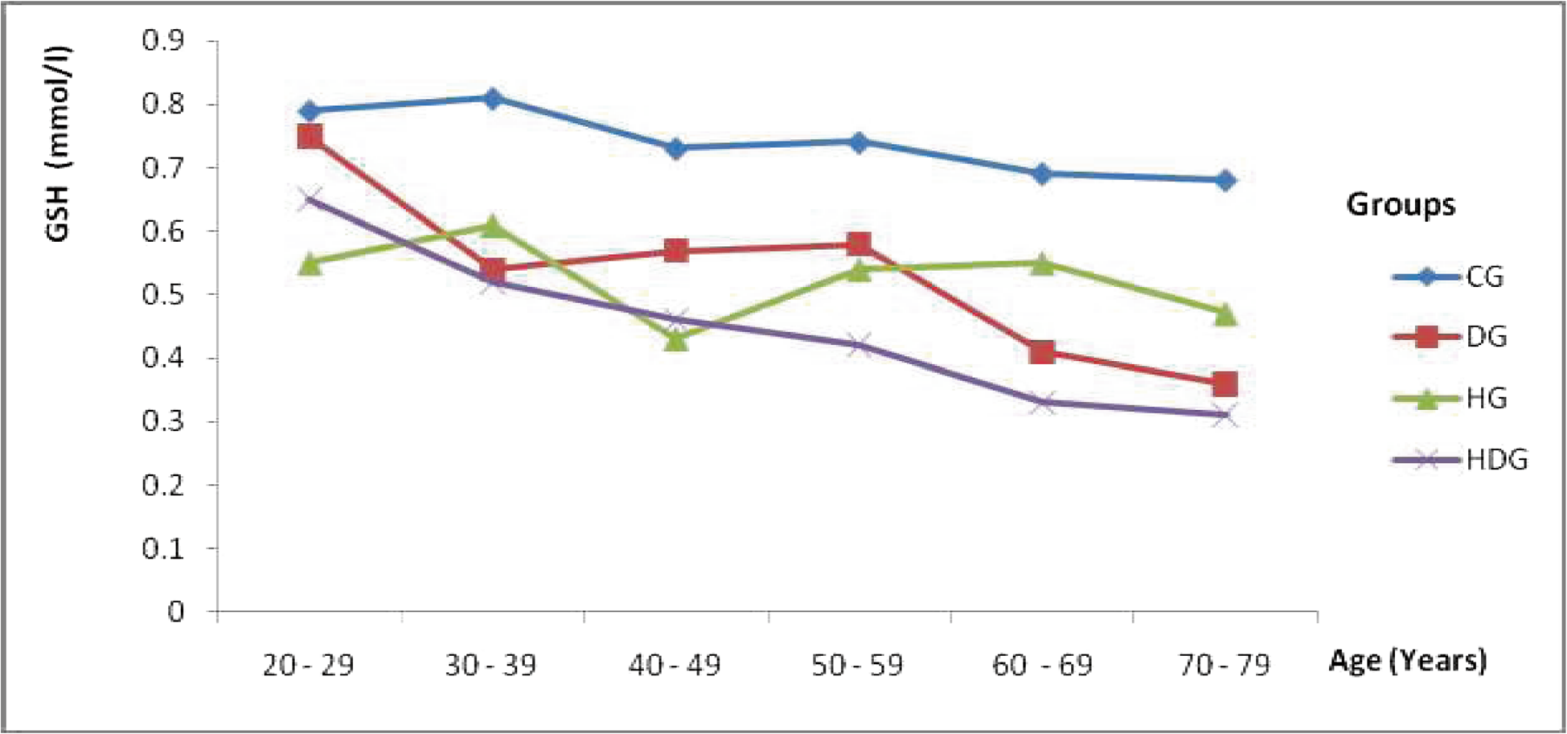
Alteration in Non-enzymatic antioxidant (GSH) levels with age. **CG**: Control Group**; DG**: Group with type 2 diabetes; **HG**: Group with hypertension. **HDG**: Group with hypertension and type-2 diabetes.

The alteration in the level of non-enzymatic antioxidant (GSH) with age was observed to be significantly (P < 0.05) higher in all the cases when compared to the control group. In all the cases there was a different trend in GSH depletion with age which was different from the control group (Figure 2). The alteration level of GSH was also observed to decrease with age between the DG and HG in a somehow similar pattern to that observed for lipid peroxidation.

## Discussion

The capability of antioxidant defense system to scavenge the damaging effect caused by ROS is determined by the contributions of GSH, antioxidative enzymes (SOD, CAT), certain vitamins (C and E) and some trace mineral elements (Se, Zn, Cu, Fe) which act as co-factors to the effectiveness in the activities of the antioxidant enzymes [4-6,9,10,13,14]. ROS like superoxide, hydrogen peroxide (H_2_O_2_), and the hydroxyl radical play a role in organ damage associated with aging and there is higher production of ROS in aged persons than young and middle-aged persons [1,8,9,11]. Excessive production of ROS is the most common cause of oxidative stress in diseases including hypertension and diabetes [4,6,17,18]. They are occasionally caused primarily by impairment of antioxidant system. In this study, the increase level of MDA observed in the case group as compared to the control group is in harmony with the results of Mahreen *et al.,* [31] and Ozdemir *et al*., [32]. The increased levels of plasma MDA reflected the lipid peroxidation as a consequence of oxidative stress. The increase in the level of the MDA is also associated with hyperglycemia in these patients, reason being that the auto-oxidation of glucose, leads to the generation of free radicals. The continual, increased in blood glucose in persons with diabetes possibly led to the generation of high levels of ROS/MDA [33-35].

Another good reason for the increased lipid peroxidation is the alteration in antioxidant defense system. For example, antioxidant protective enzymes like SOD, CAT and GPx plays a role in protection against lipid peroxidation. In this study, we observed a severe decrease in the activities of these enzymes in group with both hypertension/diabetes which became more severe as the age increases. Under normal circumstances, free radical-scavenging enzymes like SOD, CAT, GPx are the first line of cellular defense against oxidative injury, decomposing superoxide and H_2_O_2_ before interacting to form the more reactive hydroxyl radical. Decrease in the activities in SOD and CAT could be due to the inactivation of the enzymes by cross-linking or due to the exhaustion of the enzymes by increased lipid peroxidation [36].

An increase in the activities of catalase was reported in persons with diabetes [34,35]. According to the reports, Kumawat, *et al.* [34] and Ceballos, *et al.* [37] explained that during aging process, the steady state concentration of H_2_O_2_ is much higher [34,37]. This could have enhanced peroxidation of poly unsaturated fatty acid (PUFA) in cell membrane, which possibly led to the lysis of erythrocytes resulting in the increase in the level of antioxidant enzymes (GPx and Catalase) observed in their study. Nevertheless, our findings although contrary to theirs but is in agreement with the reports of many other researchers who reported decrease in antioxidant enzymes including CAT and an increase in lipid peroxidation [31,32,38,39]. H_2_O_2_ is mainly detoxified by GPx and glutathione reductase (GR) in erythrocytes. When the activity of GPx decreases due to non-availability of NADPH and GSH, the second enzyme catalases rises and disposes off the H_2_O_2_. In 2003, Kumar *et al.,* reported that due to decreased activity of GPx, there is compensatory boost in the activities of catalase [40]. So, it is obvious that the depletion of catalase activities observed in this study could be due to increased production of H_2_O_2_. Moreover, an increase in the SOD activity may protect CAT against enzyme inactivation by superoxide radical as these radicals have been shown to inactivate CAT. For that reason, increase in SOD activity may in some way play a significant protective role in conserving the activity of CAT. Our findings of decreased activities of CAT and SOD in persons with diabetes, is in conformity with that of other researchers [4,38,39]. The accumulation of H_2_O_2_ may result in several deleterious effects in disease condition [41]. The decrease in activity of SOD with increasing age group observed in the present study, reflects tissue injury due to accumulation of superoxide (O.^- 2^) radicals. The complications of diabetes may be the result of this elevated level of alteration in oxidative stress status and the reduction in antioxidant defenses.

Antioxidant vitamins C and E as well as GSH play an excellent role in protecting the cells from oxidative damage [42]. It is well-known that GSH in blood maintain the cellular levels of the active forms of Vitamin C and Vitamin E by scavenging the free radicals. Vitamins C and E are intimately related to low level of GSH in the cell [34,35,42]. This report is in agreement with our findings of decreased levels of GSH, Vitamin C and Vitamin E in the case group when compared with the control group. The decrease in the GSH level may also be interlinked with the low level of glutathione peroxidase (GPx) activities, as indicated by Selenium: its cofactor [32].

The mean serum level of Se observed in this study is in agreement with the research carried out on selenium in persons with diabetes by other research scientists [43-45]. Serum Se is a key component of a number of functional selenoproteins like GPx, which reduces H_2_O_2_ and other organic peroxides to nontoxic substances. Hence the reduced levels of this trace element may potentially lead to reduced activity of GPx and decreased antioxidant defense mechanisms. Result of several studies demonstrated that overproduction of peroxides along with emaciation of antioxidant defense system cause oxidative damage and these events in persons with type 2 diabetes are observed earlier before complication of diabetes developed [31,32,34,35]. Diplock *et al.* [46]*,* reported that when complications of diabetes developed, an increase in oxidative damage and subsequently emaciation of antioxidant defense system are observed. In diabetes, there occur glycation of glutathione peroxidase and thus functional changes of the antioxidant enzymes. Consequently decreasing antioxidant status of this selenium dependent enzyme leads to more free radical production and more complication of diabetes such as hypertension in diabetes.

An increase in serum Fe was observed in group with hypertension, which is in agreement with the finding of other researchers [35,47,48]. This increase was not significant when compared to the CG. However it is pertinent to know that excess Fe can lead to tissue damage by producing the generation of ROS. Fe^2+^ is capable of supporting lipid peroxidation, acceleration of atherosclerosis development has been postulated as a potential mechanism by which iron overload may increase the risk of ischemic cardiovascular events. Iron may also have deleterious effects on vascular function [31].

This study also showed that the levels of Zn and Se decreased in the blood of both persons with type 2 diabetes and hypertension. Serum Fe decreased in diabetes and hypertensive-diabtetic group. The loss of these antioxidant elements in diabetes might be attributed to impaired absorption and/or the excess excretion of these elements in urine (glycosuria) in these patients, which may induce a deficiency or marginal state of these elements in blood of persons with diabetes [49]. The increase in the Cu^2+^ levels in patients with diabetes might be attributed to hyperglycemia that may stimulate glycation and release of Cu^2+^ from copper-containing enzymes. This is supported by Lin [49] who reported the elevation of the concentrations of both lenticular copper ions and the protein-unconjugated copper ions than that of protein-conjugated copper ions. This results in the decrease of the reactivity of copper-containing enzymes such as SOD of persons with diabetes [33,49]. Zn plays an important role in the synthesis and function of insulin, it is capable of modulating insulin action, and it improves hepatic binding of insulin [35]. As an antioxidant, Zn has membrane-stabilizing properties and is said to preserve endothelial function because of its ability to inhibit the pathways of processes leading to apoptosis, probably by upregulating caspase genes. Serum Zn and Cu, both form the prosthetic group of SOD, any alteration in their levels affect the activity of the enzyme. Decrease in this element impairs the enzyme activity. Such a condition may cause oxidative stress or may further increase an existing stress [7,48]. Most of the case studies by other researchers have shown high Cu levels in patients with coronary artery disease, hypertension and Myocardial infarction compared to healthy controls [43,48,49]. In this study a significantly increased Cu levels was observed in the group with hypertension.

Mimic *et al.* [50] reported that depletion of GSH by 20% to 30% can impair the cell defense against the toxic action of xenobiotic and may lead to cell injury/death. In this study, we observed a depletion of GSH by 19.1%, 17.6% and 35.3% in diabetes, hypertensive and hypertensive-diabetic group respectively with respect to the control group (Table 3). The depletion was observed to increase with increasing age (Figure 2). The activities of the antioxidant enzymes were also observed too to have decreased in all the case groups (Table 3). It is well known that low activity of these enzymes render the tissue more susceptible to lipid peroxidation damage. We observed a significant increase of over 89.9% in lipid peroxidation (MDA) level in the case groups when compared to the mean value of 2.94 ± 0.05 nmol/ml as reference obtained from the control group. This observation is in accordance with the hypothesis that lipid peroxidation and glutathione peroxidase might play a role in tissue damage [50].The alteration in the activities of the antioxidant enzymes and the increased production of lipid peroxidation is directly related to increasing age. However, we observed that the percentage rate of alteration of the antioxidant system was greatly affected by the diseases: diabetes and/or hypertension.

## Conclusion

This result of the present study reveals the percentage level of alteration in antioxidants and serum trace mineral elements in persons with hypertension and/or diabetes in relation to their ages. The data provides good background information for clinicians, research workers, paramedical personnel, nutritionists and health care personnel who are working in the field of diabetes especially for disease management in this geographical location. Although we are aware of the limited population size of the present study, the major findings of this work is that a different pattern/condition of antioxidant defense system was seen in patients with hypertension and/or diabetes of varied age range. The condition might be due to an age-linked altered metabolism. To this effect, we recommend the need to establish age-dependent reference values of oxidative stress markers, since, changes in the associations determine the level of alteration in antioxidant defense system and the pathophysiologic mechanisms underlying the complication of type-2 diabetes and associated hypertension. Finally, ameliorating oxidative stress through antioxidants supplements may be useful in the management of diabetes and its associated hypertension during aging.

## Abbreviation

AAS: Atomic Absorption Spectrophotometer
ABU: Ahmadu Bello University
ABUTH: Ahmadu Bello University Teaching Hospital
ADA: American Diabetes Association
ANOVA: Analysis of Variance
CAT: Catalase
CG: Control Group
Cu: Serum Copper
DBP: Diastolic Blood Pressure
DG: Diabetic Group
DMRT: Duncan Multiple Range Test
ECDCDM: Expert Committee on the Diagnosis and Classification of Diabetes Mellitus
Fe: Serum Iron
FPG: Fasting Plasma Glucose
GPx: Glutathione Peroxidase
GSH: Reduced Glutathione
HDG: Hypertension/Diabetes Group
HG: Hypertensive Group
H_2_O_2_: Hydrogen Peroxide
MDA: Malondialdehyde
NADPH: Reduced Nicotinamide Adenine Dinucleotide Phosphate
PUFA: Poly Unsaturated Fatty Acid
ROS: Reactive Oxygen Species
SBP: Systolic Blood Pressure
Se: Serum Selenium
SOD: Superxide Dismutase
SPSS: Statistical Package for the Social Sciences
Vit-C: Vitamin C (Ascorbic Acid)
Vit-E: Vitamin E (α-Tocopherol)
WHO: World Health Organisation
WMA: World Medical Association
Zn: Serum Zinc
